# Impact of Social Support and Self-Efficacy on Activity of Daily Living among Post-Stroke Patients in Saudi Arabia: A Cross-Sectional Study

**DOI:** 10.3390/healthcare12161564

**Published:** 2024-08-07

**Authors:** Ahmed Nahari, Ali Matoug Alsaleh

**Affiliations:** 1 Medical Surgical Department, College of Nursing, King Saud University, Riyadh 11362, Saudi Arabia; 2 National Neuroscience Institute, King Fahad Medical City, Riyadh 11525, Saudi Arabia; amalsaleh@kfmc.med.sa

**Keywords:** post-stroke, activity of daily living, social support, self-efficacy, Saudi Arabia

## Abstract

This study employed a cross-sectional design to explore the impact of social support and self-efficacy on activity of daily living (ADL) among post-stroke patients in Saudi Arabia and investigate the mediating role of self-efficacy. Data were collected from 158 post-stroke patients across six healthcare facilities in three regions of Saudi Arabia using convenience sampling, between February 2023 and July 2023. The analysis included descriptive statistics, variance analysis, and linear regression using bootstrap methods. PROCESS Macro was used for the mediation model. This study revealed that most participants had high ADL, social support, and self-efficacy levels. Significant negative associations were found between ADL and age (*p* < 0.001), time since stroke (*p* = 0.009), and stroke history (*p* < 0.001), while significant positive associations were observed with educational background (*p* = 0.049), employment status (*p* < 0.001), and self-efficacy (*p* < 0.001). ADL in post-stroke patients was significantly influenced negatively by age (*p* = 0.025), time since stroke (*p* = 0.027), and stroke history (*p* < 0.001), while self-efficacy (*p* < 0.001) had a positive impact and moderated the relationship between social support and ADL. This study highlights the physical and psychosocial aspects affecting post-stroke patients, identifies key areas for enhancing their experiences, and informs the development of targeted interventions to address their comprehensive needs.

## 1. Introduction

Stroke is the second leading cause of mortality and the third leading cause of death and disability worldwide [1]. Stroke has been overwhelming financially, costing more than 700 billion dollars globally as a result of its increased incidence, prevalence, and mortality rates, which increased by 70%, 102%, and 43%, respectively, from 1990 to 2019 [1]. Globally, approximately 12 million new cases of stroke are reported each year, and one in four people aged over 25 years will experience a stroke in their lifetime [1]. In the United States of America (U.S.A), approximately 795,000 people experience stroke each year [2]. The highest rates of overall stroke-related mortality have been reported in most Asian regions (Central, Southeast, and East Asia), Oceania, and sub-Saharan Africa [1]. The number of stroke cases in the Kingdom of Saudi Arabia (KSA) is increasing yearly [3]. The annual number of cases in the Kingdom of Saudi Arabia (KSA) is estimated to be approximately 50 per 100,000 people. There are large discrepancies in the reported stroke incidence rates in the KSA. Current evidence indicates that the annual incidence rates range from 15.1 to 57.6 per 100,000 people in the KSA [4]. A 12.8% increase in the number of stroke cases is estimated over the next 10 years [5]. Additionally, evidence has shown that individuals in KSA tend to have their first stroke at a younger age compared to other countries in the Middle East, as well as Western countries such as the United States and the United Kingdom [3,4,5].

Pathophysiological changes resulting from stroke can lead to various health-related complications, including physical, mental, social, and psychological [2]. Evidence has shown that activities of daily living (ADL) are commonly impaired in post-stroke patients. ADL refers to numerous tasks performed daily, such as eating, grooming, using the toilet, and transferring [6]. Alterations in the ability to perform tasks among post-stroke patients increase the risk of dependency, with approximately 75% of post-stroke patients experiencing this challenge [7]. Furthermore, post-stroke patients commonly face excretory challenges, including urinary and bowel incontinence, with approximately 25% of patients experiencing exacerbated urinary incontinence and 56% experiencing bowel incontinence, which can affect their self-esteem and potentially lead to further complications [8,9]. Evidence supports the notion that post-stroke rehabilitation helps to significantly improve ADL and prevent poor prognosis [10].

Post-stroke patients also face psychosocial challenges, including depression and mood changes [8]. Previous research has shown that three months after stroke, post-stroke patients often experience psychological symptoms such as fatigue and cognitive impairment, which contribute to higher levels of anxiety and depression [11]. Additionally, post-stroke patients experience significant social difficulties such as returning to work, reintegrating into society, and engaging in ADL, all of which are closely tied to maintaining good relationships with family and friends [12].

Given the above-mentioned complications experienced by post-stroke patients, social support can be considered an essential element for improving their quality of life, especially during the rehabilitation phase. Social support entails an individual’s perception and access to a network of social resources for guidance, validation, and decision-making support in life experiences. Strong social support has been associated with favorable patient outcomes, improved physical function, increased participation in social and leisure activities, and ultimately, enhanced quality of life [13,14,15]. In addition, self-efficacy can play a vital role in achieving favorable outcomes in post-stroke patients. Self-efficacy, a key component of Bandura’s social cognitive theory, refers to an individual’s mental belief in their own capability to perform a specific task, encompassing both personal attributes and environmental influences [16,17]. Recent evidence has revealed that self-efficacy serves as a significant marker of quality of life in patients with chronic illnesses and is correlated with favorable outcomes in post-stroke patients [18,19].

Although physical and psychosocial aspects are vital in post-stroke patients’ experiences, little is known about ADL, social support, and self-efficacy among post-stroke patients in Saudi Arabia. Thus, this study aimed to investigate (1) the relationships between patients’ ADL and social support, self-efficacy, and sociodemographic factors; (2) the impact of social support and self-efficacy on ADL; and (3) the mediating effect of self-efficacy on the relationship between social support and ADL.

### Theoretical Framework

This study followed Bandura’s self-efficacy theory. According to Bandura’s theory, self-efficacy—the confidence in one’s ability to perform a given task—can be enhanced through social influences, including social support, which leads to greater confidence in daily activities [20]. This reciprocal relationship forms a cycle in which high levels of self-efficacy facilitate engagement in daily tasks, reinforced by ongoing social support. Thus, social support enables the development and maintenance of self-efficacy, facilitating successful engagement in daily activities [20].

## 2. Materials and Methods

Using a cross-sectional design, this study was conducted in the stroke prevention clinics of six healthcare facilities, specifically selected because they are the largest and provide the most comprehensive stroke services in their respective regions (Eastern, Central, and Western) of Saudi Arabia. These clinics are part of governmental healthcare facilities, serving stroke patients and providing various stroke services, including urgent care and follow-up visits after a stroke event, regardless of whether the patients underwent rehabilitation. These clinics provide stroke services through stroke expert clinics that contain well-equipped utilities. Data were collected using a nonprobability convenience sampling approach collected between February 2023 and July 2023. The study inclusion criteria included post-stroke patients aged 18 and above attending stroke prevention clinics at selected healthcare facilities, who had experienced a stroke within the past 2 to 60 months and had the ability to understand and complete the questionnaire. Participants with altered levels of consciousness, aphasia, or cognition were excluded. The sample size was estimated using the G*Power software (version 3.1). The power analysis indicated that a minimum of 124 participants would be required to achieve a statistical power of ≥80%, assuming a type I error rate of 0.05, a medium effect size f2 of 0.15, and 5% of missing data.

The study adhered to the Strengthening the Reporting of Observational Studies in Epidemiology (STROBE) guidelines for cross-sectional studies (Supplementary File S1).

This study included a two-part survey. The first part comprised sociodemographic data (including age, sex, marital status, educational level, religion, total income, date of last stroke event, and history of stroke). The second part contained the following three measures: (1) activities of daily living (ADL). The Arabic and validated version of ADL by Nasser and Doumit (2009) [21] was used to measure the ability to perform routine daily activities. Katz et al. (1963) developed the original ADL scale [22]. The ADL measures the level of dependence when performing six daily functions (bathing, dressing, using the toilet, transferring movements, continence, eating, and drinking). It comprises six items that measure six daily functions. The possible responses were coded as 0 (very dependent), 0.5 (partially dependent), or 1 (totally independent). The total score was obtained by summing the scores of the six items, dividing the number obtained by six, and then multiplying it by 100, with higher scores indicating higher independence [21]. The instrument’s reliability and validity have been established [21,22,23]. Reliability, as measured by Cronbach’s alpha, was 0.93 in our study. (2) Multidimensional Scale for Perceived Social Support (MSPSS): the Arabic version of this tool was used to measure social support. It is a self-report tool used widely to assess the social support in community and mental health settings and the perceived adequacy of three social support sources (family, friends, and significant others). It comprises 12 items, with a minimum score of 12 and a maximum score of 84. Lower scores indicated lower levels of social support. The psychometric properties of MSPSS have been previously reported [24]. The Cronbach α was 0.96 in our study. (3) Self-Efficacy for Managing Chronic Disease 6-items (SEMCD-6): the Arabic version of this tool was used to measure self-efficacy. The questionnaire measures people’s confidence in their ability to manage fatigue, discomfort, pain, emotional distress, and any other symptoms associated with managing chronic conditions. It comprises six items rated on a 10-point Likert scale, with a higher mean score over four out of six items indicating greater self-efficacy. The SEMCD-6 has been widely used in research and clinical practice with established validity and reliability [25]. In this study, the Cronbach α was 0.95.

Participants who met the inclusion criteria in the targeted facilities were invited to participate in the study. Those who agreed to participate were instructed to complete an electronic survey using a tablet provided by the second author. To ensure patient privacy, the second author coordinated with the facilities to provide designated rooms in which the patients could sit and complete the survey.

Data were analyzed using the Statistical Package for the Social Sciences (SPSS) version 29. Descriptive measures (average, frequency, mean, and standard deviation) were calculated for the sociodemographic data. For analysis purposes, some variables (marital status: single vs. married; educational background: high school or lower level of education vs. university or higher level of education; income: 5000 Saudi Riyals or less vs. more than 5000 Saudi Riyals; time of stroke: 12 months or less vs. more than 12-month, history of stroke occurrence: 1st-time stroke vs. 2 or more) were recorded.

Pearson’s correlation coefficient (*r*) was used to explore the associations between ADL and social support, self-efficacy, and continuous sociodemographic factors. A point-biserial correlation coefficient was used to determine the relationship between ADL and dichotomous sociodemographic variables. Multiple linear regression analysis was conducted to explore the factors affecting ADL. Furthermore, the direct effect of self-efficacy on the relationship between social support and activities of daily living in the presence of self-efficacy as a mediator was examined using regression analyses with PROCESS Macro model 4 and bootstrapping proposed by Hayes (2022) [26].

## 3. Results

### 3.1. Sample Characteristics

A total of 179 patients were approached to participate in the study. Of these, 158 patients agreed to participate and were included in the final analysis, resulting in a participation rate of 88%. As shown in Table 1, the average age was 54.47 years (SD ± 16.5). Approximately 66% of the participants were aged 45 years and above. The majority of participants were male (*n* = 103; 65.2%). Most participants (*n* = 127; 80.4%) were married, compared to single participants (*n* = 31; 19.6%). Most participants (*n* = 113; 71.5%) had a high school education or lower, whereas the remainder had a university education or higher (*n* = 45; 28.5%).

More than half of the participants were unemployed (*n* = 93, 58.9%), whereas the remaining participants were employed (*n* = 65, 41.1%). The mean history of stroke occurrence was 12.75 months (SD ± 10.2), with most participants (*n* = 103; 65.2%) having had a stroke within a period of 12 months or less. First-time stroke onset was reported by most participants (*n* = 124; 78.5%), with a mean of 1.37 stroke events (SD of 1.06). Participants lived in three regions in Saudi Arabia, with the majority living in the central region (*n* = 73; 46.2%), followed by the Eastern region (*n* = 50; 31.6%) and the Western region (*n* = 35; 22.2%).

As shown in Table 2, the mean ADL score was 80.85 (SD ± 26.5), with the majority of the participants (*n* = 96; 60.8%) categorized as totally independent, followed by partially dependent (*n* = 58; 36.7%), and then very dependent (*n* = 4; 2.5%). The MSPSS total score mean was 5.85 (SD ± 1.36), and the majority of the participants (*n* = 132; 83.5%) had high social support, followed by moderate social support (*n* = 15; 9.5%), and then low social support (*n* = 11; 7.0%). The highest source of support was from significant others (mean = 6.09; SD ± 1.45), followed by family (mean = 6.06; SD ± 1.37), and then friends (mean = 5.39; SD ± 1.64). The mean score for SEMCD was 7.79 (SD ± 2.05). The mean scores for SEMCD subscales were as follows: medication 7.92 (SD ±2.26), activity 7.85 (SD ± 2.26), physical comfort and pain 7.79 (SD ± 2.3), fatigue 7.76 (SD ± 2.28), symptoms 7.73 (SD ± 2.36), and emotional distress 7.65 (SD ± 2.43).

### 3.2. Relationships between Activity of Daily Living and Sociodemographic Factors, Social Support, and Self-Efficacy

As shown in Table 3, ADL was significantly negatively associated with age in years (r = −0.368, *p* < 0.001), indicating that participants with higher age had lower ADL levels. ADL was significantly positively associated with educational background (r = 0.156, *p* = 0.049) and with employment status (r = 0.281, *p* < 0.001). ADL was significantly negatively associated with the time of stroke (r = −0.208, *p* = 0.009) and with a history of stroke (r = −0.421, *p* < 0.001).

When exploring the correlations between ADL, social support, and self-efficacy, the analysis showed a positive and significant correlation between ADL and self-efficacy (r = 0.487, *p* < 0.001). This suggests that participants with higher ADL scores had higher perceived self-efficacy in managing the disease. However, the results indicate that social support was not correlated with ADL (r = 0.093; *p* = 0.247).

### 3.3. Factors Influencing Activity of Daily Living

A regression model was used to explore possible predictors of ADL. As shown in Table 4, the model was significant, F(10, 147) = 10.952, *p* < 0.001, explaining approximately 42.7% of the variance in ADL (R^2^ = 0.427). Significant predictors include age, with each additional year associated with a significant decrease in ADL (B = −0.289, β = −0.180, t = −2.265, *p* = 0.025, 95% CI [−0.541, −0.037]). Time since stroke was also significant, with participants who had their stroke more than 12 months ago showing worse performance in ADL compared to those who had a stroke 12 months ago or less (B = −8.173, β = −0.147, t = −2.238, *p* = 0.027, 95% CI [−15.391, −0.955]). Further, the history of stroke occurrence was a significant predictor for ADL; participants experiencing their first stroke event showed better ADL compared to those who had two or more stroke events (B = −17.726, β = −0.275, t = −4.177, *p* < 0.001, 95% CI [−26.113, −9.339]). Finally, SEMCD was a strong positive predictor for ADL (B = 4.836, β = 0.374, t = 5.284, *p* < 0.001, 95% CI [3.027, 6.645]), indicating that as participants’ belief in their perceived ability to manage their chronic disease increased, their ability to perform ADL increased. It should be noted that the remaining sociodemographic factors did not significantly impact ADL, as shown in Table 4. These factors include sex, marital status, educational background, employment, income, and MSPSS, all with *p* > 0.05.

### 3.4. The Mediating Role of Self-Efficacy on the Relationship between Social Support and Activity of Daily Living

**Figure 1:**
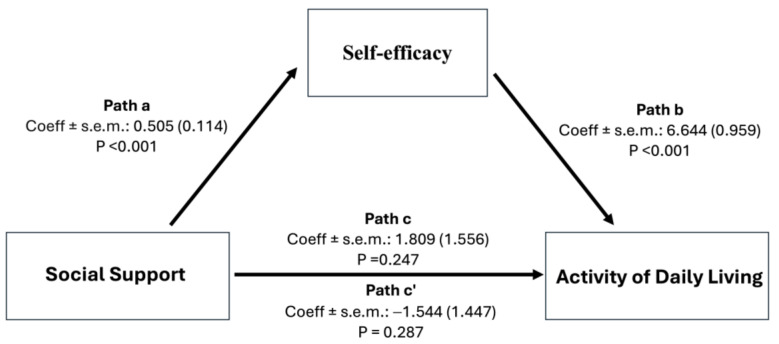
Conceptual framework of the study and the mediation role of self-efficacy on the relationship between social support and activity of daily living.

Mediation analysis was conducted to evaluate the effect of self-efficacy on the relationship between social support and ADL with self-efficacy acting as a mediator. As shown in Table 5 and Figure 1, the results indicate that while the indirect effects through self-efficacy were significant (a path: B = 0.505, *p* < 0.001; b path: B = 6.644, *p* < 0.001), the direct effect and total effect of social support on ADL were not statistically significant (c’ path: B = −1.544, *p* = 0.287; c path: B = 1.809, *p* = 0.247) with the proportion of mediation (Pm) = 1.8535, suggesting that self-efficacy fully mediated the effect of social support on ADL.

## 4. Discussion

This study explored ADL, social support, and self-efficacy in post-stroke patients, and investigated the factors affecting their ADL and self-efficacy. The findings revealed that most participants had high ADL scores. A possible explanation for this finding may be attributed to the fact that most of the participants were middle-aged. The mean age in our study was 54.47 years, which is relatively consistent with what has been reported in other studies exploring stroke among patients in Saudi Arabia [3,4,5,27]. Previous evidence has shown that individuals in Saudi Arabia tend to have their first stroke at a younger age compared to those in other Western countries [3,4,5]. This trend can be attributed to the KSA population pyramid, which is skewed towards a younger age group, rather than solely to lifestyle factors influencing stroke risk. This demographic context could have contributed to the high ADL scores reported in our study, as evidence suggests that younger individuals tend to have better functional abilities and greater independence in activities of daily living compared to older individuals [6,28,29,30,31,32]. Another possible explanation is that the majority of the patients had experienced a stroke within the past 12 months and reported it as their first stroke. Previous studies have reported that these factors are associated with higher ADLs in post-stroke patients [6,28,29,30,31,32]. In addition, our findings indicated that the majority of participants had high levels of social support and self-efficacy, which could have contributed to increased levels of ADL [15,17,33]. Therefore, it is crucial to establish initiatives that support post-stroke patients’ engagement and active interactions with friends and society. Additionally, efforts should focus on enhancing self-efficacy as a means of encouraging ADLs in post-stroke patients.

The findings of the bivariate analysis provide valuable insights into the links between ADLs and non-modifiable and potentially modifiable personal factors in post-stroke patients. Our study showed that non-modifiable factors, including age, time of stroke, and history of stroke, were associated with ADLs. Consistent with previous reports, our findings indicate that older age is associated with decreased ADLs, highlighting the decline in the functional ability of older populations. Moreover, these results are similar to those of previous studies [6,28,29,30]. The findings showed that increased duration since stroke onset and a history of multiple stroke occurrences were associated with declines in ADLs.

The findings showed that increased duration since stroke onset was associated with declines in ADLs. While this trend is typically observed in long-term chronic evaluations, our findings suggested that even among patients who experienced strokes within the past two years, those with longer durations since onset exhibited declines in ADLs. It should be noted here that the mean duration since stroke onset in our study was 12.75 months (SD ± 10.2). Our findings may be influenced by the specific characteristics of our study population, including differences in access to rehabilitation services and support systems. Evidence has shown that the majority of post-stroke patients in Saudi Arabia receive treatment in non-specialized stroke facilities, highlighting the limited availability of stroke care services at the national level [34]. A similar finding was observed in terms of the decline in ADLs in post-patients with a history of multiple stroke occurrences.

The findings revealed associations between ADLs and potentially modifiable factors, including employment status and educational level, suggesting that higher levels of education and employment are associated with increased ADLs. These findings are consistent with those of previous studies [28,35]. Although some of these factors are non-modifiable and others are not readily alterable in the short term, our findings emphasize the necessity of accounting for such factors when exploring post-stroke patients’ experiences.

Additionally, our findings indicate that ADL is associated with self-efficacy, suggesting that participants with higher ADL scores have higher perceived self-efficacy. This result is consistent with those of other studies [36], confirming a strong association between ADL and self-efficacy. Post-stroke patients with higher ADL scores tend to have higher perceived self-efficacy, indicating that their confidence in managing daily tasks positively influences their functional independence. Although previous evidence supports the link between ADL and social support [37], our study revealed conflicting results. Our findings showed a lack of association between these two variables, even though the majority of participants exhibited higher levels of both ADL and perceived social support. One possible explanation for such findings could be the improved self-sufficiency of participants in performing ADL, as it facilitated the performance of ADLs more effectively than merely having support networks available to assist with ADL-specific tasks and activities [14]. Another possible explanation may be the increased availability of post-stroke services such as rehabilitation, which could have led the participants to rely less on external support networks because of the existence of professional assistance, thus potentially obscuring the direct relationship between ADL and social support. Moreover, the participants’ differences in coping strategies or resilience levels may have mediated the link between social support and ADL, leading to variability in the strength of the association. Additionally, the quality, timing, and duration of social support may have mitigated the direct relationship between ADL and social support, as evidence has shown that these aspects of social support play a crucial role in an individual’s ability to function independently [38,39]. Our findings highlight the need for further exploration of the factors that may underlie this complex relationship.

The multivariate analysis conducted in this study revealed several significant factors affecting ADL in post-stroke patients. These findings offer valuable insights into different aspects of functional abilities. Our results suggest that age plays a crucial role in shaping ADL performance among post-stroke patients. This finding aligns with previous research [30], which emphasized that aging leads to compromised bodily functions and difficulties in resuming regular activities. As age is considered a non-modifiable factor, early interventions and programs aimed at optimizing preferred post-stroke outcomes should employ tailored strategies to mitigate the impact of aging on recovery. Inconsistent with previous evidence [40], our study found that sex did not significantly impact ADL, suggesting that both male and female post-stroke patients experience similar levels of functional independence. Such findings warrant further investigations to explore the lack of sex-based differences. Our results also showed that increased duration since stroke onset contributed to a decline in ADL. Higher rates of ADL dependence and mobility impairment have been reported in post-stroke patients, with increased duration since stroke onset [30]. Evidence suggests that despite receiving early post-stroke rehabilitation services and engaging in physical regimens, post-stroke patients are at increased risk of experiencing a gradual decline in ADL over time due to factors associated with inadequate self-care practices, insufficient family support, lack of follow-up, and lifestyle changes related to aging [6]. Similarly, our results indicated that the recurrence of stroke events has contributed to a decline in ADL. Evidence has shown that patients with multiple strokes tend to have increased damage to brain tissues, which can result in more dependence and impaired physical ability. Further, patients experiencing multiple strokes often exhibit exacerbated damage to brain tissues, which can increase their ADL dependency and worsen physical limitations [41]. The present study provides additional evidence regarding the importance of early assessment and raising awareness to prevent stroke and its complications, as well as to identify the risk factors contributing to stroke recurrence. Moreover, future research is needed to explore strategies that can enhance independence among post-stroke patients with similar long-term consequences.

Furthermore, multivariate analysis suggested that self-efficacy is a crucial determinant for improving ADL in post-stroke patients. This is consistent with previous studies reporting that higher levels of self-efficacy foster better ADL performance [17,42]. Building on this finding, our study supports the notion that interventions aimed at enhancing self-efficacy levels in post-stroke patients may lead to better functional outcomes, which in turn can contribute to improved quality of life and health outcomes [19]. Additionally, given the dynamic nature of self-efficacy, incorporating strategies aimed at strengthening key sources of self-efficacy into long-term care plans could potentially yield long-lasting results for ADL performance [20,43]. Further studies are required to establish the impact of these methods on long-term outcomes in post-stroke patients.

This study further explored the mediating role of self-efficacy in the relationship between social support and ADL. Our findings highlight the pivotal role of self-efficacy as a mediator in the relationship between social support and ADL in post-stroke patients. In contrast to our initial hypotheses and previous research [14,44], no direct effect of social support on ADL was observed, indicating that social support alone may not be sufficient to enhance ADL. Instead, our analysis suggests that the benefits of social support for ADL are primarily manifested through the enhancement of perceived self-efficacy. Despite this, our findings highlight the importance of self-efficacy in the pathway to functional outcomes, consistent with previous research highlighting the mediating role of self-efficacy in ADL among stroke patients [45,46,47]. Our findings also align with the theoretical lens underpinning our study, which suggests that an individual’s belief in their ability to achieve specific outcomes plays a critical role in shaping behavioral outcomes [48]. According to Bandura’s theory, social influence, as part of social persuasion, significantly contributes to shaping individuals’ beliefs about their capabilities through interactions and feedback from their social environment, ultimately leading to greater confidence in performing ADL. Therefore, our findings not only provide empirical evidence for the theoretical framework proposed by Bandura, but also provide important insights for future practical efforts to enhance the functional abilities of post-stroke patients, suggesting that strategies should specifically target and enhance self-efficacy within the context of providing social support. Future research should explore the specific types of social support that most effectively enhance self-efficacy and, hypothetically, improve functional abilities among post-stroke patients.

### Limitations

This study has several limitations. First, in this study, we used a convenience sampling method due to practical considerations such as accessibility and cost-effectiveness. The study was conducted in stroke prevention clinics of six government hospitals across three regions (Eastern, Central, and Western) of Saudi Arabia, selected because they were major healthcare providers with well-established stroke clinics and a high volume of stroke patients, ensuring efficient data collection and robust analysis. Future studies should consider including additional sites across a broader range of healthcare facilities nationwide to enhance the generalizability of the findings and address potential selection biases. Also, future research should employ more rigorous sampling techniques to validate these findings and explore additional factors influencing ADL in post-stroke patients. Second, the data were collected using self-reported measures, which could have introduced bias. Specifically, social desirability bias may have led to an overestimation of reported social support, self-efficacy, and ADL levels. Therefore, future studies should use alternative methodologies to mitigate the impact of social desirability bias. Third, the utilization of ADL as the primary measure of disability in this study may not fully capture the extent of patients’ functional and neurological deficits. Future research should incorporate additional measures, including detailed neurological evaluations, to provide a more comprehensive assessment of patient outcomes. Fourth, in this study, patients with altered levels of consciousness, aphasia, or cognition problems were excluded, which may have resulted in a sample that is not fully representative of the broader post-stroke population. Future studies should consider including these patients to enhance the generalizability of the findings. Finally, this study was restricted to examining specific factors that may influence ADL in post-stroke patients. The exclusion of detailed clinical variables, such as stroke type and neurological deficits at hospital discharge, was a limitation. Future research should address these factors to better understand their relationship with functional dependence and explore additional variables that could act as confounders or contributors to the functional abilities of this population.

## 5. Conclusions

This study explored ADL, social support, and self-efficacy in post-stroke patients in Saudi Arabia. Most participants in this study demonstrated higher levels of ADL, social support, and self-efficacy. Several sociodemographic factors were associated with ADL: Age, time since stroke, and history of stroke were negatively associated, while educational background and employment status were positively associated. These findings suggest that ADL among post-stroke patients in Saudi Arabia is significantly influenced by self-efficacy, age, time since stroke, and history of stroke occurrence. However, the patient population in this study predominantly consisted of patients who were middle-aged, had experienced a stroke within the past 12 months, and reported it as their first stroke, which should be considered when interpreting the results. Further studies are recommended to explore additional factors and validate these findings in broader and more diverse patient populations to support clinical practice. Additionally, this study indicated that self-efficacy plays a crucial role as a mediator in the relationship between social support and ADL in post-stroke patients. Our findings highlight the physical and psychosocial aspects that could affect the experiences of post-stroke patients in Saudi Arabia. These insights are particularly valuable for developing targeted interventions and support systems tailored to the specific cultural and social context of Saudi Arabia. Such tailored approaches can better address the comprehensive needs of post-stroke patients in Saudi Arabia, ultimately enhancing their post-stroke experiences and overall well-being.

## Figures and Tables

**Table 1:** Distribution of the sample characteristics N = (158).

Characteristics	Mean± SD or *n* (%)
Age	54.47 ± 16.5
Sex	
Male	103 (65.2%)
Female	55 (34.8%)
Marital Status	
Single	31 (19.6%)
Married	127 (80.4%)
Education level	
High school or lower level of education	113 (71.5%)
University or higher level of education	45 (28.5%)
Employment status	
Unemployed	93 (58.9%)
Employed	65 (41.1%)
Estimated income	
5000 Saudi Riyals or less	70 (44.3%)
More than 5000 Saudi Riyals	88 (55.7%)
Region	
Central	73 (46.2%)
Eastern	50 (31.6%)
Western	35 (22.2%)
Time of stroke (months)	12.75 ± 10.2
12 and less	103 (65.2%)
More than 12	55 (34.8%)
History of stroke	1.37 ± 1.06
1st time	124 (78.5%)
2 or more	34 (21.5%)

**Table 2:** Distribution of the instruments N = (158).

Instruments	Mean ± SD or *n* (%)
Activity of daily living (ADL)	80.85 ± 26.5
Very dependent	4 (2.5%)
Partially dependent	58 (36.7%)
Totally independent	96 (60.8%)
Bathing	
Very dependent	15 (9.5%)
Partially dependent	36 (22.8)
Totally independent	107 (67.7%)
Dressing	
Very dependent	14 (8.9%)
Partially dependent	44 (27.8%)
Totally independent	100 (63.3%)
Toileting	
Very dependent	16 (10.1%)
Partially dependent	29 (18.4%)
Totally independent	113 (71.5%)
Transferring	
Very dependent	7 (4.4%)
Partially dependent	46 (29.1%)
Totally independent	105 (66.5%)
Continence	
Very dependent	8 (5.1%)
Partially dependent	37 (23.4%)
Totally independent	113 (71.5%)
Feeding	
Very dependent	7 (4.4%)
Partially dependent	37 (23.4%)
Totally independent	114 (72.2%)
Multidimensional Scale of Perceived Social Support (MSPSS)	5.85 ± 1.36
Low social support	11 (7.0%)
Moderate social support	15 (9.5%)
High social support	132 (83.5%)
Source of support	
Significant others	6.09 ± 1.45
Family	6.06 ± 1.37
Friends	5.39 ± 1.64
Self-Efficacy Managing Chronic Disease (SEMCD)	7.79 ± 2.05
Fatigue	7.76 ± 2.28
Pain and physical discomfort	7.79 ± 2.23
Emotional distress	7.65 ± 2.43
Symptoms	7.73 ± 2.36
Activity	7.85 ± 2.26
Medication	7.92 ± 2.26

**Table 3:** Correlation between ADL, MSPSS, SEMCD, and sociodemographic data (N = 158).

Variables	1	2	3	4	5	6	7	8	9	10	11
1. Age in years	-										
2. Sex ^a^	−0.182 *	-									
3. Marital status ^b^	0.181 *	−0.308 **	-								
4. Education level ^c^	−0.379 **	0.157 *	−0.041	-							
5. Employment ^d^	−0.457 **	−0.125	0.057	0.441 **	-						
6. Income ^e^	−0.155	−0.311 **	0.137	0.365 **	0.538 **	-					
7. Time of stroke ^f^	0.163 *	−0.060	0.093	0.069	0.010	−0.070	-				
8. History of stroke ^g^	0.211 **	−0.027	−0.052	−0.092	−0.219 **	−0.091	0.070	-			
9. ADL score	−0.368 **	0.068	0.059	0.156 *	0.281 **	0.137	−0.208 **	−0.421 **	-		
10. MSPSS score	0.095	−0.073	−0.007	−0.087	0.105	0.065	0.129	0.030	0.093	-	
11. SEMCD score	−0.192 *	0.075	0.017	0.013	0.144	0.133	−0.080	−0.200 *	0.487 **	0.334 **	-

Note: ADL: activity of daily living; MSPSS: multidimensional scale of perceived social support; SEMCD: Self-Efficacy Managing Chronic Disease. ^a^ Sex = male vs. female; ^b^ marital status = single vs. married; ^c^ education level = high school or lower level of education vs. university or higher level of education; ^d^ employment = unemployed vs. employed; ^e^ income = 1–5000 vs. 5001 and more; ^f^ time of stroke = 12 months and less vs. more than 12 months; ^g^ History of stroke = 1st-time stroke vs. 2 or more. * *p*-value ≤ 0.05, ** *p*-value ≤ 0.01.

**Table 4:** Regression analysis of activity of daily living.

	Activity of Daily Living					
Variables					95% CI	
	B ^a^	β ^b^	t	*p*	LL	UL
Age	−0.289	−0.180	−2.265	0.025	−0.541	−0.037
Sex	0.343	0.006	0.084	0.933	−7.697	8.383
Marital status	6.083	0.091	1.357	0.177	−2.774	14.940
Educational background	3.134	0.053	0.690	0.492	−5.847	12.116
Employment	4.584	0.085	0.974	0.332	−4.719	13.887
Income	−2.804	−0.053	−0.639	0.524	−11.479	5.871
Time of stroke	−8.173	−0.147	−2.238	0.027	−15.391	−0.955
History of stroke occurrence	−17.726	−0.275	−4.177	<0.001	−26.113	−9.339
MSPSS	0.238	0.012	0.175	0.861	−2.448	2.925
SEMCD	4.836	0.374	5.284	<0.001	3.027	6.645
Model summary: F (10, 147) = 10.952, R^2^ = 0.427, *p* < 0.001						

Note: MSPSS: Multidimensional Scale of Perceived Social Support, SEMCD: Self-Efficacy Managing Chronic Disease. B ^a^ = unstandardized coefficient, β ^b^ = standardized coefficient beta, t = t-statistics, CI = confidence interval, LL = lower limit, UL = upper limit.

**Table 5:** Results of mediating effect of self-efficacy by bootstrapping (N = 158).

						95% CI		
Effect	Variables	B	SE	t	*p*-Value	LLCI	ULCI	
Direct effect (c’ path)	Social support → activity of daily living	−1.544	1.447	−1.067	0.287	−4.403	1.314	
Indirect effects (a path)	Social support → self-efficacy	0.505	0.114	4.432	0.000	0.280	0.730	
Indirect effects (b path)	Self-efficacy → activity of daily living	6.644	0.959	6.931	0.000	4.750	8.538	P_m_ = 1.8535
Indirect effects (ab path)	Social support → self-efficacy → activity of daily living	3.353	1.363	-	-	1.168	6.412	
Total effect (c path)	Social support → activity of daily living	1.809	1.556	1.163	0.247	−1.264	4.882	

Note: 5000 bootstrapping re-extracted. Abbreviations: B = B coefficient, CI = confidence interval, LLCI = lower limit of B in 95% CI, SE = standard of error, ULCI = upper limit of B in 95% CI.

## Data Availability

The data presented in this study are available on request from the corresponding author. The data are not publicly available due to privacy or ethical restrictions.

## Supplementary Materials

The following supporting information can be downloaded at: https://www.mdpi.com/article/10.3390/healthcare12161564/s1.

## References

[1] Feigin V.L., Brainin M., Norrving B., Martins S., Sacco R.L., Hacke W., Fisher M., Pandian J., Lindsay P. (2022). World Stroke Organization (WSO): Global Stroke Fact Sheet 2022. Int. J. Stroke.

[2] Tsao C.W., Aday A.W., Almarzooq Z.I., Alonso A., Beaton A.Z., Bittencourt M.S., Boehme A.K., Buxton A.E., Carson A.P., Commodore-Mensah Y. (2022). Heart Disease and Stroke Statistics—2022 Update: A Report From the American Heart Association. Circulation. Circulation.

[3] Basri R., Issrani R., Hua G.S., Prabhu N., Khursheed A.M. (2021). Burden of stroke in the Kingdom of Saudi Arabia: A soaring epidemic. Saudi Pharm. J..

[4] Alqahtani B.A., Alenazi A.M., Hoover J.C., Alshehri M.M., Alghamdi M.S., Osailan A.M., Khunti K. (2020). Incidence of stroke among Saudi population: A systematic review and meta-analysis. Neurol. Sci..

[5] Al-Senani F., Al-Johani M., Salawati M., Alhazzani A., Morgenstern L.B., Ravest V.S., Cuche M., Eggington S. (2020). An Epidemiological Model for First Stroke in Saudi Arabia. J. Stroke Cerebrovasc. Dis..

[6] Wurzinger H., Abzhandadze T., Rafsten L., Sunnerhagen K.S. (2021). Dependency in Activities of Daily Living During the First Year After Stroke. Frontiers in Neurology. Front. Neurol..

[7] Kim K., Kim Y.M., Kim E.K. (2014). Correlation between the Activities of Daily Living of Stroke Patients in a Community Setting and Their Quality of Life. J. Phys. Ther. Sci..

[8] Chohan S.A., Venkatesh P.K., How C.H. (2019). Long-term complications of stroke and secondary prevention: An overview for primary care physicians. Singap. Med. J..

[9] Jacob L., Kostev K. (2020). Urinary and fecal incontinence in stroke survivors followed in general practice: A retrospective cohort study. Ann. Phys. Rehabil. Med..

[10] Tani T., Imai S., Fushimi K. (2022). Rehabilitation of Patients With Acute Ischemic Stroke Who Required Assistance Before Hospitalization Contributes to Improvement in Activities of Daily Living: A Nationwide Database Cohort Study. Arch. Rehabil. Res. Clin. Transl..

[11] Khazaal W., Taliani M., Boutros C., Abou-Abbas L., Hosseini H., Salameh P., Sadier N.S. (2021). Psychological Complications at 3 Months Following Stroke: Prevalence and Correlates Among Stroke Survivors in Lebanon. Front. Psychol..

[12] Lehnerer S., Hotter B., Padberg I., Knispel P., Remstedt D., Liebenau A., Grittner U., Wellwood I., Meisel A., BSA Long Term Care Study Group (2019). Social work support and unmet social needs in life after stroke: A cross-sectional exploratory study. BMC Neurol..

[13] Glass T.A., Matchar D.B., Belyea M., Feussner J.R. (1993). Impact of social support on outcome in first stroke. Stroke.

[14] Elloker T., Rhoda A.J. (2018). The relationship between social support and participation in stroke: A systematic review. Afr. J. Disabil..

[15] Alshahrani A.M. (2020). Quality of life and social support: Perspectives of Saudi Arabian stroke survivors. Sci. Progress.

[16] Barnett A., Michalos A.C. (2014). Self-Efficacy. Encyclopedia of Quality of Life and Well-Being Research.

[17] Gangwani R., Cain A., Collins A., Cassidy J.M. (2022). Leveraging Factors of Self-Efficacy and Motivation to Optimize Stroke Recovery. Front. Neurol..

[18] Peters M., Potter C.M., Kelly L., Fitzpatrick R. (2019). Self-efficacy and health-related quality of life: A cross-sectional study of primary care patients with multi-morbidity. Health Qual. Life Outcomes.

[19] Szczepańska-Gieracha J., Mazurek J. (2020). The Role of Self-Efficacy in the Recovery Process of Stroke Survivors. Psychol. Res. Behav. Manag..

[20] Bandura A. (1977). Self-efficacy: Toward a unifying theory of behavioral change. Psychol. Rev..

[21] Nasser R., Doumit J. (2009). Validity and reliability of the Arabic version of Activities of Daily Living (ADL). BMC Geriatr..

[22] Katz S., Ford A.B., Moskowitz R.W., Jackson B.A., Jaffe M.W. (1963). Studies of Illness in the Aged: The Index of ADL: A Standardized Measure of Biological and Psychosocial Function. J. Am. Med. Assoc..

[23] Nasser R., Doumit J., Al-Attiyah A., Fokhroo H. (2013). Effect of Belief in a Just World on Daily Living Activities of Nursing Home Residents. Soc. Behav. Personal. Int. J..

[24] Merhi R., Kazarian S. (2012). Validation of the Arabic translation of the Multidensional Scale of Social Support (Arabic MSPSS) in a Lebanese community sample. Arab. J. Psychiatry.

[25] Allam M.M., El-Zawawy H.T., Ibrahim Ismail I., Ghazy R.M. (2020). Cross-Cultural Reliability of an Arabic Version of the Self-Efficacy for Managing Chronic Disease 6-Item Scale in Arab Patients with Diabetes mellitus. Prim. Care Diabetes.

[26] Hayes A. (2022). Introduction to Mediation, Moderation, and Conditional Process Analysis A Regression-Based Approach.

[27] Alfakeeh F.K., Alghamdi Y.M., Alharbi B.F., Alotaibi A.M., Alsaleh K.A., Alshubaili A.M., Mcrabi R.H., Alenazi F.K., Almuklass A. (2024). HbA1c and risk factors’ prevalence in patients with stroke: A retrospective study in a tertiary care hospital in Saudi Arabia. Neurosci. J..

[28] Edemekong P.F., Bomgaars D.L., Sukumaran S., Schoo C. (2024). Activities of Daily Living. StatPearls.

[29] Ismail N.R., Hamid A.A., Razak A.A., Hamid N.A.A. (2021). Factors Influencing Instrumental Activities of Daily Living (IADL) Disability among Elderly Attending Health Clinics in Kelantan. Malays. Appl. Biol..

[30] Shao C., Wang Y., Gou H., Chen T. (2024). The factors associated with the deterioration of activities of daily life in stroke patients: A retrospective cohort study. Top. Stroke Rehabil..

[31] Gao J., Gao Q., Huo L., Yang J. (2022). Impaired Activity of Daily Living Status of the Older Adults and Its Influencing Factors: A Cross-Sectional Study. Int. J. Environ. Res. Public Health.

[32] Whitiana G.D., Vitriana V., Cahyani A. (2017). Level of Activity Daily Living in Post Stroke Patients. Althea Med. J..

[33] Lee Y., Won M. (2022). Mediating Effects of Rehabilitation Motivation between Social Support and Health-Related Quality of Life among Patients with Stroke. Int. J. Environ. Res. Public Health.

[34] Temehy B., Soundy A., Sahely A., Palejwala Y., Heath J., Rosewilliam S. (2023). Exploring the needs of stroke patients after discharge from rehabilitation centres in Saudi Arabian communities: An IPA qualitative exploratory study design. PLoS ONE.

[35] Finan J.M., Landes S.D. (2024). Educational Attainment and Perceived Need for Future ADL Assistance. J. Appl. Gerontol..

[36] Pompey C.S., Muensri B., Kritpracha C. (2016). Self-Efficacy to Perform Activities of Daily Living Predicts Independence in Activities of Daily Living in Subacute Stroke Patients. GSTF J. Nurs. Health Care (JNHC).

[37] Bozo O., Toksabay N.E., Kürüm O. (2009). Activities of daily living, depression, and social support among elderly Turkish people. J. Psychol..

[38] Tariq A., Beihai T., Abbas N., Ali S., Yao W., Imran M. (2020). Role of Perceived Social Support on the Association between Physical Disability and Symptoms of Depression in Senior Citizens of Pakistan. Int. J. Environ. Res. Public Health.

[39] Drageset J., Haugan G., Eriksson M. (2021). Social Support. Health Promotion in Health Care–Vital Theories and Research.

[40] Liljehult M., von Euler-Chelpin M., Christensen T., Buus L., Stokholm J., Rosthøj S. (2021). Sex differences in independence in activities of daily living early in stroke rehabilitation. Brain Behav..

[41] Pei L., Zang X.Y., Wang Y., Chai Q.W., Wang J.Y., Sun C.Y., Zhang Q. (2016). Factors associated with activities of daily living among the disabled elders with stroke. Int. J. Nurs. Sci..

[42] Kim J.H., Park E.Y. (2013). Balance self-efficacy in relation to balance and activities of daily living in community residents with stroke. Disabil. Rehabil..

[43] Liu W., Galik E., Resnick B. (2015). The Self-Efficacy for Functional Abilities Scale for Older Adults in Long-Term Care: Two-Level Exploratory and Confirmatory Factor Analysis. J. Nurs. Meas..

[44] Han S., Shan L.B., Wang G.X., Ke Y.Z., Meng S.Q., Li Y.X., Cui Z.L., Tong W.X. (2022). Physical Fitness, Exercise Behaviors, and Sense of Self-Efficacy Among College Students: A Descriptive Correlational Study. Front. Psychol..

[45] French M.A., Moore M.F., Pohlig R., Reisman D. (2016). Self-efficacy mediates the relationship between balance/walking performance, activity, and participation after stroke. Top Stroke Rehabi..

[46] Nott M., Wiseman L., Seymour T., Pike S., Cuming T., Wall G. (2021). Stroke self-management and the role of self-efficacy. Disabil. Rehabil..

[47] Oh S. (2020). How future work self affects self-efficacy mechanisms in novel task performance: Applying the anchoring heuristic under uncertainty. Personal. Individ. Differ..

[48] Cudicio A., Agosti V. (2024). Beyond Belief: Exploring the Alignment of Self-Efficacy, Self-Prediction, Self-Perception, and Actual Performance Measurement in a Squat Jump Performance—A Pilot Study. J. Funct. Morphol. Kinesiol..

